# Effects of Cherry Consumption on Metabolic Health: A Pilot Clinical Study on Healthy Adults

**DOI:** 10.3390/ijms26188891

**Published:** 2025-09-12

**Authors:** Filomena Carvalho, Alexandra Varges, Radhia Aitfella Lahlou, Eduardo Bárbara, Isa Santos, Cecília Fonseca, Luís R. Silva

**Affiliations:** 1 SPRINT–Sport Physical Activity and Health Research & Innovation Center, Polytechnic of Guarda, 6300-559 Guarda, Portugal; 2 Family Health Unit–Carolina Beatriz Ângelo, Local Health Unit of Guarda, 6300-035 Guarda, Portugal; 3 Family Health Unit–A Ribeirinha, Local Health Unit of Guarda, 6300-690 Guarda, Portugal; 4 IPG, Polytechnic of Guarda, 6300-559 Guarda, Portugal; 5 CMA–Center of Mathematics and Applications, University of Beira Interior, 6201-506 Covilhã, Portugal; 6 RISE-Health, Faculty of Health Sciences, University of Beira Interior, 6201-506 Covilhã, Portugal; 7 CERES, Department of Chemical Engineering, University of Coimbra, 3030-790 Coimbra, Portugal

**Keywords:** cherry consumption, metabolic health, phenolic compounds, anti-inflammatory, antioxidant

## Abstract

Cherry consumption has been associated with several metabolic health benefits, due to their rich profile of bioactive compounds, including anthocyanins. This pilot clinical study, which is, to our knowledge, the first evaluating consumption of whole cherries, aimed to evaluate the effects of daily cherry consumption on oxidative stress, inflammation, glycaemic regulation, and other metabolic health markers in healthy adults. A total of 27 volunteers consumed 280 g of sweet cherries daily for 42 days, followed by a two-week post-intervention period. Significant improvements were observed in glucose regulation, including reduced HbA1c and estimated average glucose levels. Markers of inflammation, such as IL-6 and AGP-1 were significantly reduced during the intervention period. In addition, reductions in GGT and LDH indicated potential hepatoprotective effects. These results suggest that regular cherry consumption may serve as a preventive strategy against early metabolic dysfunction, highlighting the need for further investigation with larger, long-term clinical trials.

## 1. Introduction

Metabolic disorders, such as obesity, insulin resistance, hypertriglyceridemia, lower HDL concentration and hypertension collectively define metabolic syndrome (MetS) [1]. MetS is a risk factor for cardiovascular diseases and type 2 diabetes (T2D) [1,2], with over 537 million adults living with diabetes alone as of 2021 [3]. According to global data from a meta-analysis, MetS currently affects from 12.5% to 31.4% of the world’s population depending on the criteria used for diagnosis [4].

Inflammation and oxidative stress are key contributors to the development of metabolic comorbidities, including hyperlipidaemia, hypertension, and impaired glucose tolerance, all of which contribute to metabolic dysfunction [5,6]. In recent years, evidence has suggested that lifestyle factors, particularly dietary habits, such as following a Mediterranean diet, physical exercise, sleep changes and stress reduction play an essential role in MetS prevention [7].

In this context, the consumption of functional foods rich in bioactive compounds has gained increasing attention as a natural approach to mitigate the risks associated with metabolic disorders. Among these, cherries (*Prunus* spp.), particularly sweet and tart varieties, are increasingly recognized for their unique phytochemical profile, which includes phenolic acids, flavonoids, mainly anthocyanins, and tannins [8]. Anthocyanins are the pigments responsible for the vibrant red-purple colour of cherries, and they have been shown to modulate oxidative stress by scavenging reactive oxygen species (ROS) and enhancing endogenous antioxidant defences [9]. These potent antioxidants have demonstrated anti-inflammatory [10,11], antidiabetic [12,13], anticancer [14,15], neuroprotective [16,17] and cardioprotective properties [18,19] in several studies.

Cherries have been linked to anti-inflammatory properties through downregulation of pro-inflammatory cytokines, such as interleukin-6 (IL-6) and tumour necrosis factor-alpha (TNF-α) [20,21]. These effects are of particular relevance, given the role of inflammation in the development of insulin resistance and beta-cell dysfunction in the pancreas, which are precursors to T2D [22,23]. Furthermore, some studies have demonstrated the ability of cherry juices to influence glycaemic markers, including fasting glucose and glycated haemoglobin (HbA1c), potentially improving insulin sensitivity and glucose metabolism [24,25].

Despite accumulating evidence for the health-promoting properties of cherries, much of the research to date has focused on populations with existing health conditions, such as arthritis [26], gout [27], hypertension [28] or MetS [29] specifically. While these studies provide valuable insights, they leave a critical gap in understanding the potential benefits of cherries in healthy individuals. Examining the effects of cherry consumption in metabolically healthy adults can help to determine its role in prevention rather than treatment, particularly for mitigating early risk factors associated with the development of metabolic disorders. In addition, and as far as we know, our clinical trial is the first to test the consumption of the whole cherry fruit, instead of preparations or extracts.

Our pilot study aims to investigate the effects of cherry consumption on oxidative stress, inflammation, glycaemic regulation and other metabolic health markers in a population of healthy adults. By exploring these metabolic and biochemical parameters, this research seeks to provide an understanding of how cherries may contribute to metabolic health and act as a preventive strategy against the progression of metabolic dysregulation.

## 2. Results and Discussion

### 2.1. Baseline Characteristics of the Participants

The study cohort consisted of 27 healthy volunteers (20 females and 7 males), aged between 18 and 64 years, with a mean age of 47 ± 12 years. The mean Body Mass Index (BMI) at baseline was 25.5 ± 3.9 kg/m^2^, ranging from 19.1 to 32.4 kg/m^2^, placing most participants within the normal to overweight category [30]. The meanwaist circumference (WC) was 86 ± 12.1 cm, which is lower than the threshold associated with increased risk of diseases (102 cm for men and 88 cm for women) [31]. The sample was predominantly composed of individuals with higher education (70.4%), with married participants accounting for 59.3% of the total sample (Table 1).

### 2.2. Metabolic Biomarkers

As the first approach, it was analysed whether significant differences existed across the four time points (baseline, Day 21, Day 42, and 2 weeks post-intervention) for each variable. Significant differences were observed for HbA1c (*p* < 0.001), estimated average glucose (*p* < 0.001), AGP-1 (*p* < 0.001), GGT (*p* = 0.010), LDH (*p* = 0.040), albumin (*p* < 0.001), LDH (*p* = 0.040), creatinine (*p* < 0.001), ferritin (*p* = 0.028), GPx (*p* < 0.001), GR (*p* < 0.001), IgG (*p* < 0.001), total IgE (*p* < 0.001), rheumatoid factor (*p* = 0.014) and ASO (*p* = 0.038) (Appendix A–Table A1). Although Friedman Test indicated no statistically significant differences across the time points in some variables, subsequently special pairwise comparisons were conducted next, with the aim of identifying potentially meaningful differences between individuals’ initial state of health and their state at different points in time, that might be masked in the overall analysis. Among the time groups, no statistically significant changes were observed in the lipid metabolism (Appendix B—Table A2) or the pancreatic function (Appendix B—Table A3) markers for any time point compared to the baseline.

#### 2.2.1. Glucose Regulation

Metabolic syndrome is characterized by high levels of glucose in the blood, therefore, maintaining optimal glucose levels is crucial for overall metabolic health [32,33]. In the present study, the impact of cherry consumption on glucose regulation markers revealed significant improvements in long-term glycaemic control (Table 2). HbA1c levels showed significant reductions at all time points, reflecting enhanced glucose management over the study duration. The estimated average glucose levels exhibited similar trends, further supporting the hypothesis of improved long-term glucose regulation. The difference of magnitude of the changes observed, with a higher decrease on day 42 compared to 2 weeks after discontinuing cherry consumption, suggest a stronger effect during active intake. This partial return to baseline values post-intervention indicates that the bioactive compounds exert their effects through regular dietary exposure.

Diabetes is characterised by hyperglycaemia, elevated glucose levels that can lead to insulin resistance, oxidative stress, and inflammation [34]. Cherries are notably rich in anthocyanins and other phenolics [35]. In recent years, phenolic compounds have shown great potential in controlling hyperglycaemia [36]. Different potential mechanisms have been described, including mechanisms in the intestine, liver, muscles, adipose tissue, and pancreatic *β*-cells, or beneficial prebiotic effects within the digestive tract [37]. Our findings suggest that dietary interventions involving cherries could be used as a complementary strategy for glycaemic management, particularly in populations at risk of developing T2D. The statistically significant reductions in HbA1c and estimated average glucose levels highlight the potential role of cherries in enhancing long-term glycaemic control.

Interestingly, while fasting glucose and insulin levels did not show statistically significant changes, the decreasing trend observed suggests a potential for improvement with a longer intervention period. It is likely that the study duration was not enough to induce detectable changes in these markers, considering the complex regulatory mechanisms involved in glucose metabolism. A prolonged or higher-dose consumption of cherries might induce more pronounced effects on fasting glucose and insulin levels, which demands further investigation in future studies.

#### 2.2.2. Inflammation and Oxidative Stress Markers

Several metabolic diseases are connected to oxidative stress and inflammation through complex mechanisms. Oxidative stress results from an imbalance between the generation of ROS and the efficiency of antioxidant defence systems [38]. Oxidative stress triggers inflammatory responses by activating redox-sensitive transcription factors, which enhance the production of proinflammatory cytokines, such as IL-6 [39]. CRP is a protein produced in the liver, which levels also increase in response to inflammation. It is primarily induced by the effect of IL-6’s on the gene that regulates CRP during the acute phase of inflammation [40]. AGP-1 is a serum glycoprotein, and its concentration is often increased during acute inflammation [41]. Chronic inflammation establishes a sustains a cycle in which oxidative stress continuously intensifies inflammatory reactions. Over time, this imbalance disrupts cellular metabolism, contributing to the progression of metabolic syndrome, T2D, obesity, and other related disorders [7,42].

Cherry consumption led to significant reductions in key inflammatory markers, such as the proinflammatory cytokine IL-6 and AGP-1 (Table 3). These markers followed the same pattern as the glucose markers, with a decrease in their concentration at all times, stronger in day 42, and starting to return to baseline levels after the intervention. Polyphenols from red fruits have been shown to reduce the expression of IL-6 in vitro, suggesting anti-inflammatory activity of these compounds [43]. A systematic review and meta-analysis on clinical trials with tart cherry, however, showed no significant changes in IL-6 levels [44]. CRP did exhibit a downward trend, however, did not show statistically significant changes in our intervention. Given that it is primarily induced by changes in IL-6, it is possible that a longer intervention period is necessary to observe significant reductions in CRP levels. To our knowledge, there is no previous research about decreased levels of AGP-1 after consumption of phenolic-rich foods. Given that this protein is one of the markers for monitoring inflammation [45], the observed reduction in its concentration following our treatment suggests that the cherries may have attenuated inflammatory processes happening within the body.

Regarding oxidation markers, a significant decrease in GPx and GR’s activity was found, particularly at day 42 for both and two weeks post-intervention for GPx (Table 4). GPx is an essential antioxidant enzyme that reduces hydrogen peroxide and lipid peroxides, protecting cells from oxidative damage [46]. GR plays a crucial role in maintaining glutathione homeostasis by regenerating reduced glutathione, a molecule with key roles in the defence against oxidative stress [47]. Given the previously reported antioxidant effects of cherries, this result may seem counterintuitive. However, the observed reductions can possibly reflect an adaptive response to lower oxidative stress, rather than a decrease in antioxidant protection. When oxidative pressure decreases, the cellular demand for GPx and GR may diminish, leading to a down-regulation of their activity: for example, in a study about the antioxidant effect of following a mediterranean diet in kidney transplant recipients, the researchers observed that GPx activity decreased after six months. They attributed this effect to a reduction in hydrogen peroxide production, the main substrate for these enzymes [48].

GDH levels remained unchanged throughout the intervention period. GDH plays a crucial role in redox balance by catalysing the oxidative deamination of glutamate to α-ketoglutarate, essential to maintain cellular energy and redox state under stress conditions [49]. Thus, cherry consumption did not significantly influence GDH-related oxidative processes. 

Overall, these findings suggest that cherry consumption may improve the antioxidant status of the body, but the mechanism behind it needs to be further clarified.

#### 2.2.3. Liver Function and Nutritional Status

Liver function plays a crucial role in overall metabolic health, influencing glucose metabolism, lipid regulation, detoxification processes, and protein synthesis [50]. In metabolic disorders, including metabolic syndrome and T2D, hepatic function is often compromised, leading to elevated levels of liver enzymes and disrupted protein metabolism [51].

The GGT levels of the volunteers demonstrated a statistically significant reduction two weeks after the intervention, suggesting a potential hepatoprotective effect of cherries (Table 5). GGT is a well-established marker for metabolic syndrome, as part of liver function tests, and is used to predict the risk of cardiovascular and cerebrovascular diseases [51]. Higher levels have been associated with cardiovascular risk in subjects with non-alcoholic fatty liver disease (NAFLD) [52,53], and with insulin resistance and T2D [54]. Phenolic compounds have been shown to mitigate oxidative damage and enhance hepatic detoxification pathways, by the modulation of different lives enzymes [55]. The delayed response in our GGT reduction, observed only after two weeks post-intervention, might indicate a cumulative or sustained effect of cherry-derived bioactive compounds on hepatic function.

LDH exhibited a significant reduction by day 42 (Table 5). LDH is involved in cellular energy metabolism, and elevated levels are often linked to liver injury, hypoxia, and systemic inflammation [56,57,58]. Thus, the observed decrease in LDH suggests that cherry consumption may contribute to a reduction in hepatic stress or systemic inflammation. Phenolic acids and flavonoids from fruit extracts have been shown to reduce carbofuran-induced hepatic damage in vivo, shown by a decrease in LDH and GGT levels, among other markers [59]. The lack of sustained significance in LDH reduction beyond day 42, however, raises questions about the duration and consistency of this effect and a longer follow-up period might be necessary.

Albumin levels consistently declined throughout the study, with statistically significant reductions at all measured time points compared to baseline (Table 5). Research remains inconclusive about the connection between albumin levels and metabolic syndrome. Studies have shown that higher levels of serum albumin are correlated with metabolic syndrome [60,61], while others report an association between higher albumin levels and lower risk of metabolic syndrome [62]. One possible explanation for the observed decrease is an adaptive response to changes in metabolic homeostasis, since this protein has a role in drug binding, inflammatory responses, and immune regulation, acting also an antioxidant [63]. However, it is proven that elevated levels show a strong correlation with increased triglycerides, high blood sugar, and excess abdominal fat [60], which aligns with our findings, where we observed a reduction in albumin levels alongside reduction in glucose levels. Nonetheless, total protein levels remained unchanged, indicating that protein synthesis was not significantly impacted. This stability further supports the notion that cherry consumption did not negatively affect protein metabolism or liver synthetic capacity.

The significant changes observed in specific liver markers, particularly GGT and LDH, suggest that cherries may exert hepatoprotective effects, potentially mediated through antioxidant and anti-inflammatory pathways. Anthocyanins have been shown to improve liver injury, by modulating key cellular pathways related to oxidative stress and inflammation, such as nuclear factor erythroid 2-related factor 2 (Nrf2) and nuclear factor-kappa B (NF-*κ*B) [64].

In contrast to the significant changes in GGT, LDH, and albumin, other liver markers, AST, ALT, and bilirubin levels (total, conjugated, and unconjugated), did not exhibit statistically significant alterations throughout the intervention. The stability of AST and ALT, primary markers for hepatocellular injury [65], suggests that cherry consumption did not induce liver damage, confirming the hepatic safety of cherries as a dietary intervention.

#### 2.2.4. Kidney Function

The kidneys play a fundamental role in the maintenance of homeostasis, through functions such as filtration of waste products, regulation of water-electrolyte balance and modulation of blood pressure [66]. Impaired kidney function has been linked to metabolic disorders [67] and, if left untreated, can lead to chronic kidney disease [68].

We observed a progressive statistically significant reduction in the creatinine levels of the individuals (Table 6). Serum creatinine is a marker used to assess kidney function, with elevated levels often indicating potential kidney dysfunction. However, since creatinine is also influenced by factors such as diet and muscle mass, this marker should be used carefully and in combination with other markers [69].

Urea is a metabolic byproduct of protein catabolism and is primarily excreted by the kidneys. It serves as an indirect indicator of renal function and protein metabolism efficiency; however, it can increase because of other factors such as starvation, low-protein diet or severe liver disease [70]. In our study, urea showed a statistically significant increase at two weeks post-intervention (Table 6).

The delayed increase of urea concentration post-intervention suggests a possible compensatory response after stopping the consumption of cherries. Cherries may have enhanced renal efficiency and reduced oxidative stress during the intervention, and their discontinuation may have led to a temporary metabolic alteration. Given that creatinine levels continued to decrease, the observed increase in urea does not necessarily indicate a decline in kidney function. Previous studies have shown that dietary polyphenols can preserve renal health and prevent damage in the kidneys by regulating mitochondrial function through regulation of the mitochondrial redox status, apoptosis, and multiple intercellular signalling pathways [71]. However, further studies are needed to explore these assumptions and understand whether prolonged cherry consumption could maintain stable urea levels over time.

The levels of microalbumin, recognised as an early marker of chronic kidney disease associated with renal function loss risk [72]; and uric acid, another marker, with elevated levels being associated with renal pathologies, such as chronic kidney disease, hypertension and acute kidney injury [73], remained unchanged through the whole study.

#### 2.2.5. Iron Metabolism

Iron is an essential trace element involved in oxygen transport, enzymatic functions, and cellular metabolism. It is strongly regulated to balance absorption, storage, and mobilization, preventing both deficiency and excess, which can lead to oxidative stress and organ dysfunction [74]. Iron metabolism disturbances have been related with diabetes, with excess iron contributing to insulin resistance, inflammation, and oxidative damage [75].

Our findings indicate a significant reduction in ferritin levels at day 42, suggesting an effect of cherry consumption on iron metabolism (Table 7). Ferritin is a key protein in the regulation of iron homeostasis and a recognized marker of iron status. Elevated ferritin levels have been linked to metabolic syndrome, T2D, and systemic inflammation, as ferritin also acts as an acute-phase reactant during inflammatory state [76,77]. The observed decrease in ferritin levels following cherry consumption may be indicative of reduced iron overload or attenuation of inflammation, similarly to what has been suggested in a previous meta-analysis, where dietary polyphenol supplementation was proved to decrease the ferritin levels of patients [78]. The polyphenol resveratrol, for example, can reduce iron overload by the reduction of iron-induced oxidative stress in rat models [79]. Flavonoids, including anthocyanins, can chelate transition metal ions, such as Fe(II) and Fe(III), reducing the availability of iron to take part in redox reactions that generate ROS and cause iron-mediated oxidative stress [80,81].

The decrease in ferritin was not maintained after the intervention period, as the values started returning to baseline two weeks post-intervention. This suggests that the regulatory effects of cherry consumption on iron metabolism could likely be dependent on continuous dietary exposure.

Although ferritin exhibited a significant reduction, other iron metabolism markers, iron, TIBC, and transferrin, did not show statistically significant changes throughout the intervention period. Serum iron reflects the amount of iron available in the bloodstream for immediate use in erythropoiesis and other metabolic processes [82], while transferrin is responsible for iron transport [83]. TIBC is used together with other tests, such as serum iron and transferrin, and helps measure the iron-binding capacity of transferrin, important for the diagnosis of iron-related disorders [84,85]. The lack of significant alterations in these markers suggests that the observed reduction in ferritin may not be due to a reduction of iron stores by cherries, causing iron deficiency, but rather linked to metabolic regulation or anti-inflammatory effects, which highlights their potential role as a dietary strategy for metabolic health.

#### 2.2.6. Immune System

The immune system plays a crucial role in maintaining homeostasis, protecting against infections, and modulating inflammatory responses [86]. Conditions such as obesity and T2D are linked to dysregulation of the immune system, where impaired immune tolerance and chronic inflammation play a crucial role. In obesity, excessive production of adipokines can reduce insulin sensitivity and contribute to development of metabolic syndrome [87,88].

Immunoglobulins (Ig), also known as antibodies, are essential components of humoral immunity, and they have different classes with different roles in immune defence and inflammatory regulation [89]. In our study, IgA levels exhibited a significant reduction at day 21, followed by a trend towards normalization (Table 8). IgA is the main antibody involved in mucosal immunity, playing a key role in preventing pathogen invasion and maintaining microbiota homeostasis [90]. This decrease could suggest changes in gut microbiota composition and reduced mucosal inflammation, leading to less need for immune activation. A study has shown that tart cherry, high in anthocyanins and polyphenols, can modulate gut microbiota in vitro, by increasing beneficial bacteria such as *Bacteroides* and *Bifidobacterium;* in the same study, an intervention in humans led to varying results, with tart cherry consumption decreasing *Bacterioides* and *Bifidobacterium* in high-*Bacterioides* individuals, and increasing them in low-*Bacterioides* individuals [91]. More clinical trials are needed to clarify these outcomes.

IgG values were significantly higher than baseline values at all time points (Table 8). IgG is the most abundant circulating immunoglobulin, with the longest half-life, and it plays a key role in immune defence by providing antibacterial and antiviral protection, neutralizing toxins and regulating immune responses [92,93]. A possible explanation for the increase in IgG levels could be an improved immunity rather than pathological immune activation, leading to a better ability of the body to respond to pathogens. A study with Jamaican cherry described a stimulation of IgG production after administration of fruit extracts in mice, suggesting enhanced immune response [94]. However, clinical research on the specific impact of cherries on IgG levels is still limited.

Regarding IgM, its levels remained stable at day 21 but showed a significant reduction at day 42, with no significant change two weeks after the intervention (Table 8). IgM is the first antibody produced in an immune response and is often elevated in acute inflammation [95]. This delayed reduction suggests that the previously mentioned ability of cherries to reduce oxidative stress and inflammation may potentially lead to a downregulation of acute immune responses, including the production of IgM.

Total IgE showed a significant reduction at all time points (Table 8). IgE plays an important role in allergic responses and is linked to inflammatory processes in conditions such as allergic rhinitis [96]. A previous study with two dietary flavones described a suppression in the total IgE levels in mice fed with two dietary flavones, due to the suppression of Th2 cytokines, suggesting the potential of this diet in managing allergic diseases [97].

Lastly, RF significantly decreased at day 21, but this effect was not sustained at later time points (Table 8). RF is an autoantibody associated with rheumatoid arthritis and other inflammatory conditions [98]. This also aligns with the hypothesis that cherries induced an improvement in immune tolerance and inflammatory activity, however, this effect was temporary, which might indicate a need for a longer exposure to cherry polyphenols to achieve more long-lasting effects.

ASO levels, which are elevated in cases of previous streptococcal infections, such as rheumatic fever [99], remained unchanged throughout the study, since participants did not experience streptococcal exposure shortly beforehand or during the intervention.

Overall, our findings suggest that cherry consumption may have beneficial immunomodulatory effects, enhancing specific humoral responses while reducing inflammatory markers, supported by previous works [100]. However, the relationship between cherry polyphenols and specific immunological markers needs to be further elucidated with additional clinical studies.

#### 2.2.7. Body Weight and Waist Circumference

Lastly, we also assessed changes in the body of the participants between the start and the end of the study, by measuring their WC, weight and height, which we then used to calculate their BMI. We did not find any significant change in the BMI of participants by the end of the study, comparing with the start (*t*-test: t = 0.217; *p* = 0.474). For the WC, we found a significant decrease in the median of the results (Wilcoxon test: Z = −2.413; *p* = 0.016). Excess visceral fat accumulation contributes to metabolic dysfunctions, including chronic low-grade inflammation, insulin resistance, and oxidative stress [101,102,103]. WC is a good anthropometric marker that assesses adiposity, and it is a better predictor of metabolic risk than BMI alone [104]. Studies have shown that reductions in WC, even in the absence of significant weight loss, are associated with improvements in metabolic health (reduced hypertension, obesity and dyslipidaemia, for example) and a lower risk of cardiovascular complications [105,106]. Emerging evidence suggests that polyphenols can modulate gut microbiota composition, promoting the establishment of beneficial probiotics and inhibiting pathogenic bacteria [107]. Polyphenols can modify microbial composition and enhance gut health [107], which could potentially lead to reductions in WC [108].

### 2.3. Participant Feedback

At the end of the study, all the participants were asked to describe any side effects that they might have experienced and their overall wellbeing through the period of cherry consumption. Out of the 27 participants, 7 reported feeling generally better than usual during the study, 4 felt significantly better, 14 stated they felt the same and 2 reported feeling worse than before the start of the consumption period. Five people reported experiencing occasional gastrointestinal symptoms, such as diarrhoea, bloating and flatulence. Two people described improved sleep quality, while 5 individuals claimed to have higher energy levels.

The reported gastrointestinal symptoms could be linked to the fact that cherries contain sorbitol, which acts as a laxative, stimulating bowel movements [109]. They are also rich in dietary fibre, which promotes a healthy gut function, leading to more regular bowel movements [110]. Although these effects might be beneficial in regulating intestinal movements and relieving constipation, the 280 g dose applied in our study may have exceeded the threshold for digestive tolerance in some cases, particularly in more sensitive individuals. Future studies should aim to identify the optimal dose that balances therapeutic benefits with gastrointestinal tolerance.

Cherries are also a natural source of melatonin, which is a hormone that regulates the sleep-wake cycle [111]. A clinical trial has shown that the consumption of tart cherries increases circulating melatonin levels, improving sleep duration and quality [112], hence the sleep quality improvement of the volunteers. Additionally, cherries have a low glycaemic index, providing a sustained source of energy without causing blood sugar spikes, which is particularly useful for endurance athletes [113]. Tart cherry juice, for example, has been proven to help in muscle recovery after intense exercise by increasing antioxidant capacity, reducing inflammation and lipid peroxidation [114]. These effects can justify the feeling of increased energy levels in some of the volunteers of our study.

## 3. Materials and Methods

### 3.1. Study Design and Participant Population

**Figure 1:**
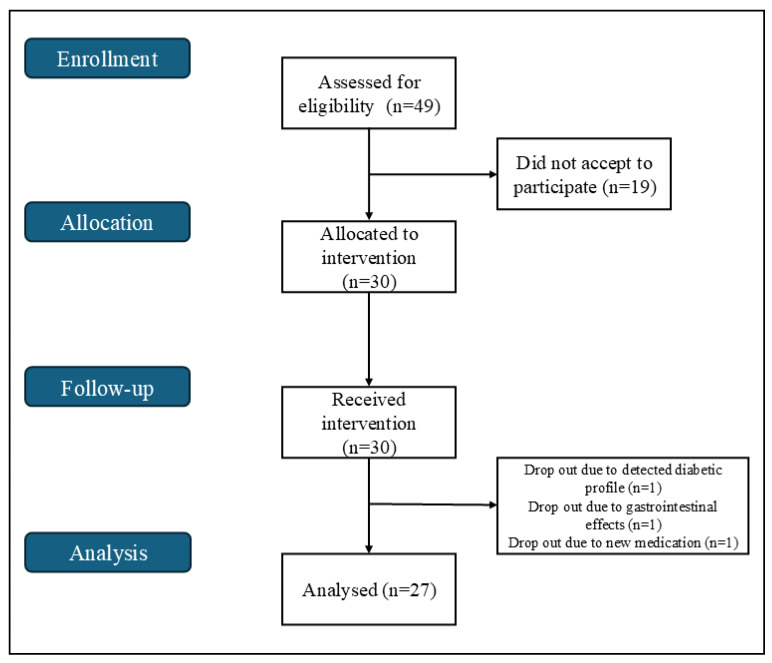
Flowchart for the single-arm trial.

Our study was designed as a longitudinal, single-arm intervention (Figure 1) and was approved by the Ethics Committee of the Local Health Unit of Guarda (document no. 88/2023). The intervention is also registered at ClinicalTrials.gov (ID: NCT07155915; date: 3 September 2025). A total of 49 volunteers were recruited among the Local Health Unit and the Polytechnic Institute of Guarda, of which a total of 27 individuals (20 woman and 7 man) aged 18–64 years old, completed the trial. Inclusion criteria for participants included being between the ages of 18 and 65 and having no known metabolic disorders. Exclusion criteria included consumption of alcohol, smoking, diagnosed chronic diseases, adverse reactions to cherry, use of antibiotics, anxiolytics or supplements, pregnancy or lactation and weight variations > 10% during the previous year.

The volunteers provided written informed consent prior to their participation. Demographic data, including age, sex, marital status, and other relevant characteristics, were collected through a pre-intervention survey. All participants’ information was anonymized to ensure confidentiality and compliance with ethical guidelines. All procedures followed the Declaration of Helsinki’s principles.

Participants were instructed to avoid the consumption of other phenolic-rich foods such as red fruits, some vegetables, chocolate, tea and red wine starting two weeks before the study and during for its duration the intervention. At this point, two weeks before the intervention, a screening blood test was performed and confirmed that none of the parameters indicated any signs of disease or health conditions.

### 3.2. Intervention

**Figure 2:**
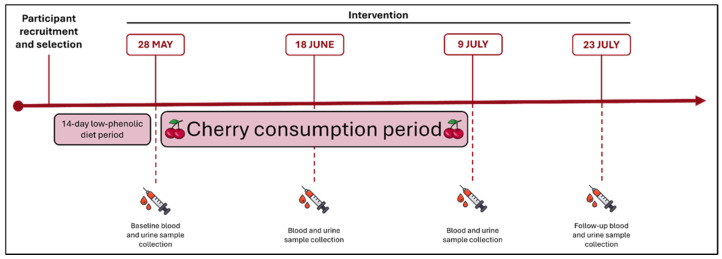
Clinical trial timeline.

On the baseline day, participants underwent their first blood and urine sample collection after an overnight fast. Following this, they started a daily consumption of 280 g of the “Prime-Giant” variety sweet cherries (*Prunus avium*) from the Fundão region, in Portugal, provided by the company CerFundão. The chemical composition of the variety has been previously analysed by the research group and is described in Table 9. The fruits were weighted, divided in daily portions and provided by the research team on a weekly basis. Participants were instructed to consume the cherries in the morning on an empty stomach for a period of 42 consecutive days (from 28 May to 9 July 2024) [24]. The cherries were kept under refrigerated conditions to ensure their freshness. Additional blood and urine sample collections were performed on day 21, day 42 (final day of intervention), and 2 weeks post-intervention (day 56), after participants ceased cherry consumption (Figure 2). Compliance with the intervention was monitored through periodic follow-up calls.

### 3.3. Sample Collection and Biochemical Analysis

All blood and urine samples were collected in the morning after an overnight fast of at least 12 h in Egianálise, a professional laboratory located in Guarda. Urine was collected by the volunteer in a urine container and saved in refrigeration conditions until analysis. Blood samples were collected by trained professionals, using vacuum tubes suitable for each specific analysis. The samples were centrifuged at 3500 rpm for 10 min to obtain serum or plasma and analysed for biochemical parameters related to overall health.

#### 3.3.1. Glucose Regulation Markers

Fasting glucose (mg/dL) and estimated average glucose (mg/dL) were determined using the hexokinase method on the Atellica IM 1600 Analyser (Siemens Healthineers, Lisbon, Portugal). HbA1c (%) was measured by high-performance liquid chromatography (HPLC) using the Bio-Rad D-10 analyser (Bio-Rad, Lisbon, Portugal). Insulin (µIU/mL) levels were quantified using the chemiluminescence method with the Siemens Immulite 2000 analyser (Siemens Healthineers, Lisbon, Portugal).

#### 3.3.2. Lipid Metabolism Markers

Triglycerides (mg/dL), total cholesterol (mg/dL), HDL cholesterol (mg/dL), and LDL cholesterol (mg/dL) were measured using enzymatic colorimetric assays, performed on the Atellica IM 1600 Analyser.

#### 3.3.3. Inflammation Markers

C-reactive protein (CRP) (mg/dL) was quantified by immunoturbidimetry, using the Atellica IM 1600 Analyser. Interleukin-6 (IL-6) (pg/mL) and alpha-1-acid glycoprotein (AGP-1) (mg/dL) were analyzed using chemiluminescent immunoassays on the Siemens Immulite 2000 analyser.

#### 3.3.4. Liver Function and Nutritional Status Markers

Aspartate aminotransferase (AST) (UI/L), alanine transaminase (ALT) (UI/L), gamma-glutamyl transferase (GGT) (UI/L), gamma-glutamyl transferase (GGT) (IU/L), total bilirubin (mg/dL), conjugated bilirubin (mg/dL), unconjugated bilirubin (mg/dL), lactate dehydrogenase (LDH) (UI/L), total proteins (g/dL) and albumin (g/dL)) were measured using enzymatic and colorimetric methods on the Atellica IM 1600 Analyser.

#### 3.3.5. Kidney Function Markers

Urea (mg/dL) and creatinine (mg/dL) were analyzed using enzymatic methods on the Atellica IM 1600 Analyser. Uric Acid (mg/dL) was measured via the uricase/peroxidase method on the same platform. Microalbumin (mg/L) was assessed in urine samples using immunoturbidimetry.

#### 3.3.6. Iron Metabolism Markers

Iron (ug/dL), total iron-binding capacity (TIBC) (µg/dL) and transferrin (mg/dL) were determined via colorimetric assays using the Atellica IM 1600 Analyser. Ferritin (ng/mL)) was quantified using chemiluminescence immunoassay with the Siemens Immulite 2000 analyser.

#### 3.3.7. Oxidative Stress Markers

Glutathione Peroxidase (GPx) (U/g prot), glutathione reductase (GR) (kU/L), glutamate dehydrogenase (GDH) (U/L) were measured using spectrophotometric methods on the Atellica IM 1600 Analyser.

#### 3.3.8. Pancreatic Function Markers

Amylase (U/L) and lipase (U/L)) were determined using enzymatic colorimetric methods on the Atellica IM 1600 Analyser.

#### 3.3.9. Immunological Markers

Immunoglobulin A (IgA) (mg/dL), immunoglobulin G (IgG) (mg/dL), immunoglobulin M (IgM) (mg/dL), total immunoglobulin E (IgE) (kU/L), rheumatoid factor (IU/mL) and antistreptolysin O (ASO) (IU/mL)) were assessed using chemiluminescent immunoassays on the Siemens Immulite 2000 analyser.

During the first and last sample collections, the WC, height and weight of the participants were measured. The height and weight of the participants were used to calculate their BMI (kg/m^2^).

### 3.4. Statistical Analysis

All statistical analyses were performed using IBM SPSS Statistics^®^ version 29.0.0.0 for Windows^®^ (New York, NY, USA). A descriptive analysis of the participants’ sociodemographic characteristics was performed. The analysis of differences across all the time points, for each variable, was performed by the Friedman test, a non-parametric alternative to repeated measures ANOVA. This test was chosen because the data did not fit the normal distribution, verified by the Shapiro-Wilk, and for its robustness for analysing small sample sizes. Upon finding a significant overall effect, we conducted post hoc pairwise comparisons to identify specific differences. To evaluate the mean differences between paired samples, of continuous variables, *t*-tests were conducted when the data fit the normal distribution and there was correlation (Pearson correlation coefficient), as it offers greater statistical power than non-parametric alternatives when assumptions are satisfied. For paired samples of continuous variables that did not meet the normality assumption, the Wilcoxon signed-rank test was applied. A significance level of *p* < 0.05 was considered statistically significant for all comparisons.

## 4. Conclusions

We believe that our study provides valuable insights into the potential metabolic benefits of cherry consumption. However, some limitations should be considered when interpreting the findings.

First, while significant changes were observed in certain biomarkers, these effects may not be sustained beyond the two-week post-intervention period. Future studies with longer duration could provide valuable insights on the chronic benefits of regular cherry consumption, particularly for individuals with pre-existing metabolic conditions such as obesity, T2D, and cardiovascular diseases. It would be interesting to evaluate whether cherries can contribute to the management of these conditions over a longer time frame, potentially reducing the need for pharmaceutical interventions.

Second, the non-random 27-participant sample size may introduce selection bias and not provide enough confidence to detect slight changes in other biomarkers, limiting the generalizability of the results. The small sample size also reduces statistical power. Some subgroups may be underrepresented/overrepresented, such as in terms of age, gender or education level. Future studies should consider larger and random samples to better understand the potential differential effects of cherry consumption in diverse demographic groups.

In the future, it is also important to determine the optimal dose and frequency of cherry consumption. While our study used a daily intake of 280 g, individual tolerance to dietary components such as sorbitol and fibre may vary. Investigating the effects of different dosages could help identify the most effective and tolerable levels for achieving the desired metabolic and hepatic benefits. Additionally, dose-response studies could help define consumption guidelines for the general population as well as for those with specific health needs.

In addition, future research should investigate the bioavailability, metabolism, and mechanisms of action of the compounds responsible for these effects (anthocyanins, flavonoids, etc.) in humans. Studies that track the absorption and bioactivity of the compounds could be helpful in determining how they influence biological pathways such as antioxidant activity, anti-inflammatory responses, and particularly gut microbiota modulation, given its importance and association with several conditions. This would allow us to identify the most effective polyphenol-rich cherry varieties and/or preparations for specific health outcomes.

Another potential limitation of our study is the self-reported feedback regarding side effects and overall well-being. Future studies could study directly the gastrointestinal function, sleep quality, and energy levels, among others. Additionally, although dietary guidelines were provided, the participants’ diets were not strictly controlled, so any changes in their eating habits could have influenced the results. There is also the possibility that not all participants strictly complied to the fasting period before providing blood samples. Physical activity levels may also have varied during the intervention period, and were not monitored, potentially affecting metabolic markers. In female participants, the phase of the menstrual cycle could also influence biochemical parameters.

Lastly, future studies should also consider the impact of cherries on other markers of oxidative stress and inflammation, particularly in conditions associated with chronic inflammatory diseases, such as rheumatoid arthritis, inflammatory bowel disease, or neurodegenerative disorders. Cherries should be analysed as therapy for managing these conditions, either alone or in combination with other therapeutic interventions.

In conclusion, regular consumption of sweet cherries significantly improves key metabolic health markers in healthy adults. Improvements were observed in glucose regulation, inflammation, antioxidant activity, liver and kidney function, and immune response, highlighting the broad systemic effects of cherry intake. Although some effects were not sustained post-intervention, the findings suggest that sustained cherry consumption could contribute to long-term metabolic health and inflammation regulation. Further research with larger cohorts and longer follow-ups is essential to confirm these results, explore underlying mechanisms and establish optimal intake.

## Figures and Tables

**Table 1:** Characteristics of the participants collected in the beginning of the study.

		Healthy Adults (*n* = 27)
**Age, **$\bar{x}$ **(range)**		47 ± 12 (18–64)
**BMI (kg/m^2^), **$\bar{x}$ **(range)**		25.5 ± 3.9 (19.1–32.4)
**WC (cm), **$\bar{x}$ **(range)**		86.0 ± 12.1 (64.0–110.0)
**Sex, *n* (%)**	Male	7 (25.9)
	Female	20 (74.1)
**Marital status, *n* (%)**	Single	7 (25.9)
	Married	16 (59.3)
	Divorced	3 (11.1)
	Widowed	1 (3.7)
**Education level, *n* (%)**	Basic	1 (3.7)
	Secondary	7 (25.9)
	Higher	19 (70.4)

$\bar{x}$ = mean (represented by mean ± standard deviation).

**Table 2:** Changes in glucose regulation markers over time, compared to baseline values.

Marker	t-Value/Z-Value (*p*-Value)		
	Day 21	Day 42	2 Weeks After Intervention
**Fasting glucose ^b^**	−0.064 (0.949)	−0.761 (0.447)	−1.308 (0.191)
**HbA1c ^a^**	−6.447 (<0.001) *	−3.864 (<0.001) *	−2.583 (0.017) *
**Estimated average glucose ^a^**	−6.453 (0.001) *	−3.844 (0.001) *	−2.597 (0.016) *
**Insulin ^b^**	−0.480 (0.631)	−0.525 (0.600)	−1.582 (0.114)

* Statistically significant (*p* < 0.05). ^a^ In these variables, the data followed a normal distribution, and the paired samples *t*-test was used, with results represented by the t-value. ^b^ In these variables, at least one time point did not meet the normality assumption; therefore, the Wilcoxon signed-rank test was applied, and results are represented by the Z-value. HbA1c = Glycated haemoglobin.

**Table 3:** Changes in inflammation markers over time, compared to baseline values.

Marker	t-Value/Z-Value (*p*-Value)		
	Day 21	Day 42	2 Weeks After Intervention
**CRP ^b^**	−0.349 (0.949)	−0.497 (0.447)	−1.049 (0.191)
**IL-6 ^b^**	−0.447 (<0.001) *	−1.826 (<0.001) *	−1.604 (0.017) *
**AGP-1 ^b^**	−8.59259 (0.001) *	−4.84 (0.001) *	−4.17391 (0.016) *

* Statistically significant (*p* < 0.05). ^b^ In these variables, at least one time point did not meet the normality assumption; therefore, the Wilcoxon signed-rank test was applied, and results are represented by the Z-value. CRP = C-reactive protein; IL-6 = Interleukin 6; AGP-1 = Alpha-1-acid glycoprotein.

**Table 4:** Oxidative stress markers over time, compared to baseline values.

Marker	t-Value/Z-Value (*p*-Value)		
	Day 21	Day 42	2 Weeks After Intervention
**GPx ^a^**	−1.833 (0.078)	−12.952 (<0.001) *	−8.918 (<0.001) *
**GR ^a^**	−0.583 (0.563)	−3.910 (<0.001) *	−2.021 (0.056)
**GDH ^b^**	−0.577 (0.564)	−0.444 (0.657)	−1.034 (0.301)

* Statistically significant (*p* < 0.05). ^a^ In these variables, the data followed a normal distribution, and the paired samples *t*-test was used, with results represented by the t-value. ^b^ In these variables, at least one time point did not meet the normality assumption; therefore, the Wilcoxon signed-rank test was applied, and results are represented by the Z-value. GPx = Gluthatione peroxidase; GR = Gluthatione reductase; GDH = Glutamate Dehydrogenase.

**Table 5:** Liver function and nutritional status markers over time, compared to baseline values.

Marker	t-Value/Z-Value (*p*-Value)		
	Day 21	Day 42	2 Weeks After Intervention
**AST ^b^**	−0.326 (0.744)	−1.347 (0.178)	−1.617 (0.106)
**ALT ^b^**	−0.064 (0.949)	−0.383 (0.701)	−0.802 (0.423)
**GGT ^b^**	−0.194 (0.846)	−1.852 (0.064)	−3.000 (0.003) *
**Total bilirubin ^b^**	−0.096 (0.923)	−0.188 (0.850)	−0.174 (0.862)
**Conjugated bilirubin ^b^**	−0.191 (0.849)	−0.624 (0.532)	−1.330 (0.183)
**Unconjugated bilirubin ^b^**	−0.180 (0.857)	−0.081 (0.936)	−0.829 (0.407)
**LDH ^b^**	−0.394 (0.694)	−2.194 (0.028) *	−1.582 (0.114)
**Total proteins ^a^**	0.591 (0.559)	−0.817 (0.422)	−1.286 (0.212)
**Albumin ^b^**	−3.322 (<0.001) *	−3.396 (<0.001) *	−3.816 (<0.001) *

* Statistically significant (*p* < 0.05). ^a^ In these variables, the data followed a normal distribution, and the paired samples *t*-test was used, with results represented by the t-value. ^b^ In these variables, at least one time point did not meet the normality assumption; therefore, the Wilcoxon signed-rank test was applied, and results are represented by the Z-value. AST = Aspartate aminotransferase; ALT = Alanine transaminase; GGT = Gamma-glutamyl transferase; LDH = Lactate dehydrogenase.

**Table 6:** Kidney function markers over time, compared to baseline values.

Marker	t-Value/Z-Value (*p*-Value)		
	Day 21	Day 42	2 Weeks After Intervention
**Urea ^a^**	0.178 (0.860)	1.145 (0.264)	3.296 (0.003) *
**Creatinine ^b^**	−2.202 (0.028) *	−2.714 (0.007) *	−3.119 (0.002) *
**Microalbumin ^b^**	−1.007 (0.314)	−1.867 (0.062)	−1.218 (0.223)
**Uric acid ^a^**	1.084 (0.288)	1.797 (0.085)	1.798 (0.086)

* Statistically significant (*p* < 0.05). ^a^ In these variables, the data followed a normal distribution, and the paired samples *t*-test was used, with results represented by the t-value. ^b^ In these variables, at least one time point did not meet the normality assumption; therefore, the Wilcoxon signed-rank test was applied, and results are represented by the Z-value.

**Table 7:** Iron metabolism markers over time, compared to baseline values.

Marker	t-Value/Z-Value (*p*-Value)		
	Day 21	Day 42	2 Weeks After Intervention
**Iron ^b^**	−0.961 (0.336)	−1.915 (0.055)	−0.152 (0.879)
**TIBC ^b^**	−0.637 (0.524)	−1.534 (0.125)	−0.624 (0.533)
**Transferrin ^b^**	−0.036 (0.971)	−1.709 (0.087)	−0.152 (0.879)
**Ferritin ^b^**	−0.086 (0.931)	−2.788 (0.005) *	−1.829 (0.067)

* Statistically significant (*p* < 0.05). ^b^ In these variables, at least one time point did not meet the normality assumption; therefore, the Wilcoxon signed-rank test was applied, and results are represented by the Z-value. TIBC = Total iron-binding capacity.

**Table 8:** Immunological markers over time, compared to baseline values.

Marker	t-Value/Z-Value (*p*-Value)		
	Day 21	Day 42	2 Weeks After Intervention
**IgA ^b^**	−2.079 (0.038) *	−1.952 (0.051)	−1.382 (0.167)
**IgG ^a^**	3.317 (0.003) *	2.247 (0.034) *	4.164 (<0.001) *
**IgM ^b^**	−0.025 (0.980)	−2.207 (0.027) *	−0.635 (0.526)
**Total IgE ^b^**	−4.253 (<0.001) *	−4.114 (<0.001) *	−3.741 (<0.001) *
**Rheumatoid factor ^b^**	−2.666 (0.008) *	−0.351 (0.726)	−1.403 (0.161)
**ASO ^b^**	−0.344 (0.731)	−1.670 (0.095)	−1.872 (0.061)

* Statistically significant (*p* < 0.05). ^a^ In these variables, the data followed a normal distribution, and the paired samples *t*-test was used, with results represented by the t-value. ^b^ In these variables, at least one time point did not meet the normality assumption; therefore, the Wilcoxon signed-rank test was applied, and results are represented by the Z-value. IgA = Immunoglobulin A; IgG = Immunoglobulin G; IgM = Immunoglobulin M; Total IgE = Total immunoglobulin E; ASO = Antistreptolysin O.

**Table 9:** Quantification of phenolic compounds found in the Prime-Giant variety of *Prunus avium* [115].

Compound	Quantification (µg/g Dried Fruit)
Feruloyl di-hexose	39.0 ± 2.18
Feruloyl hexose	93.5 ± 2.90
Protocatechuic acid derivative	57.3 ± 1.63
Coumaroyl hexose derivative	4.1 ± 0.20
3-*O*-caffeoylquinic acid	274.5 ± 8.54
Caffeoylquinic acid-glycoside 2	68.7 ± 1.18
3-Caffeoylquinic acid *cis*	19.8 ± 0.43
Procyanidin dimer B type 1	132.3 ± 4.05
3-Coumaroylquinic acid *trans*	8.3 ± 0.38
3-Coumaroylquinic acid *cis*	32.0 ± 0.91
Caffeoyl hexose	3.1 ± 0.06
5-Caffeoylquinic acid *trans*	40.5 ± 1.88
Taxifolin rutinoside	7.7 ± 0.10
Quercetin 7-*O*-glucoside-3-*O*-rutinoside	3.7 ± 0.46
Quercetin 3-*O*-rutinoside	3.6 ± 0.32
Quercetin 3-*O*-hexoside	9.3 ± 0.13
3,5-Dicaffeoylquinic acid	0.4 ± 0.18
Quercetin derivative	5.6 ± 0.72
Total	803.4
Anthocyanins	
Cyanidin 3-*O*rutinoside	3.6 ± 0.071
Pelargonidin 3-*O*-rutinoside	0.9 ± 0.13
Peonidin 3-*O*rutinoside	0.1 ± 0.01
Total	4.6

Quantities are expressed as mean ± standard deviation.

## Data Availability

The original contributions presented in this study are included in the article. Further inquiries can be directed to the corresponding author.

## Appendix A

**Table A1:** Results of the Friedman test assessing differences across the four time points (baseline, Day 21, Day 42, and 2 weeks post-intervention) for all markers.

	Chi-Square	df	*p*-Value
**Fasting glucose**	1.133	3	0.769
**HbA1c**	30.948	3	<0.001 *
**Estimated average glucose**	30.948	3	<0.001 *
**Insulin**	7.435	3	0.059
**Triglycerides**	1.633	3	0.652
**Total Cholesterol**	6.956	3	0.073
**HDL Cholesterol**	5.365	3	0.147
**LDL Cholesterol**	3.776	3	0.287
**CRP**	1.154	3	0.764
**IL-6**	3.333	3	0.343
**AGP-1**	20.088	3	<0.001 *
**AST**	1.679	3	0.642
**ALT**	2.069	3	0.558
**GGT**	11.296	3	0.010 *
**Total Bilirubin**	0.658	3	0.883
**Conjugated bilirubin**	4.814	3	0.186
**Unconjugated bilirubin**	1.271	3	0.736
**LDH**	8.300	3	0.040 *
**Total proteins**	4.108	3	0.250
**Albumin**	35.580	3	<0.001 *
**Urea**	6.781	3	0.079
**Creatinine**	17.979	3	<0.001 *
**Microalbumin**	7.612	3	0.055
**Uric acid**	2.823	3	0.420
**Iron**	2.868	3	0.412
**TIBC**	6.642	3	0.084
**Transferrin**	6.485	3	0.090
**Ferritin**	9.068	3	0.028 *
**GPx**	51.783	3	<0.001 *
**GR**	18.926	3	<0.001 *
**GDH**	0.652	3	0.884
**Amylase**	5.095	3	0.165
**Lipase**	5.593	3	0.133
**IgA**	7.266	3	0.064
**IgG**	17.073	3	<0.001 *
**IgM**	4.577	3	0.205
**Total IgE**	30.252	3	<0.001 *
**Rheumatoid factor**	10.680	3	0.014 *
**ASO**	8.453	3	0.038 *

* Statistically significant (*p* < 0.05). df = degreed of freedom; HbA1c = Glycated haemoglobin; CRP = C-reactive protein; IL-6 = Interleukin 6; AGP-1 = Alpha-1-acid glycoprotein; AST = Aspartate aminotransferase; ALT = Alanine transaminase; GGT = Gamma-glutamyl transferase; LDH = Lactate dehydrogenase; TIBC = Total iron-binding capacity; GPx = Gluthatione peroxidase; GR = Gluthatione reductase; GDH = Glutamate Dehydrogenase; IgA = Immunoglobulin A; IgG = Immunoglobulin G; IgM = Immunoglobulin M; Total IgE = Total immunoglobulin E; ASO = Antistreptolysin O.

## Appendix B

### Appendix B.1

**Table A2:** Changes in lipid metabolism markers over time, compared to baseline values.

Marker	t-Value/Z-Value (*p*-Value)		
	Day 21	Day 42	2 Weeks After Intervention
**Triglycerides ^b^**	−0.613 (0.540)	−0.529 (0.597)	−0.091 (0.927)
**Total Cholesterol ^a^**	−1.267 (0.216)	−1.642 (0.114)	1.318 (0.201)
**HDL Cholesterol ^a^**	−0.453 (0.654)	−0.333 (0.742)	1.720 (0.099)
**LDL Cholesterol ^b^**	−1.283 (0.200)	−1.529 (0.126)	−1.126 (0.260)

^a^ In these variables, the data followed a normal distribution, and the paired samples *t*-test was used, with results represented by the t-value. ^b^ In these variables, at least one time point did not meet the normality assumption; therefore, the Wilcoxon signed-rank test was applied, and results are represented by the Z-value.

### Appendix B.2

**Table A3:** Pancreatic function markers over time, compared to baseline values.

Marker	t-Value/Z-Value (*p*-Value)		
	Day 21	Day 42	2 Weeks After Intervention
**Amylase ^b^**	−0.601 (0.548)	−0.872 (0.383)	−1.446 (0.148)
**Lipase ^b^**	−0.529 (0.597)	−1.458 (0.145)	−1.332 (0.183)

^b^ In these variables, at least one time point did not meet the normality assumption; therefore, the Wilcoxon signed-rank test was applied, and results are represented by the Z-value.
